# Supplementary material for: Analysis of the Associations between Arthritis and Fall Histories in Korean Adults
Source: Int J Environ Res Public Health. 2021 Apr 3;18(7):3758. doi: 10.3390/ijerph18073758 (PMC8038444; doi:10.3390/ijerph18073758)
Supplement: Supplementary file 1 [file ijerph-18-03758-s001.pdf]

Supplement file of the description of The Korean Community Health Survey (KCHS)

This study was the cross-sectional study using the data from the KCHS. The data covered one nation using statistical methods based on designed sampling and adjusted weighted value. The KCHS conducted in 2015 and 2017 were analyzed. The survey gathered information through face-to-face, paper assisted personal interviews between trained interviewers and respondents. The 2015 and 2017 KCHS data were based on 254 communities and conducted by KCDC, 17 metropolitan cities, 254 community health centers, and 35 community universities and committees. The KCHS data used a two-stage sampling process clustered sampling. The first stage selected a sample area (tong/ban/ri) as a primary sample unit, which was selected according to the number of households in the area using a probability proportional to the sampling method. In the second stage, the number of households in the selected sample tong/ban/ri was identified to create a household directory. Sample households were selected using systematic sampling methods. This process was applied to ensure that the sample units were representative of the entire population. For the sample to be statistically representative of the population, the data collected from the survey were weighted by statisticians who performed post-stratification and considered the non-response rates.

Supplement file of the description of other covariates

To measure physical activity, the participants were asked about the number of days they engaged in vigorous exercise with considerable shortness of breath for ≥ 10 minutes in the last week and the number of days they engaged in moderate exercise with slight shortness of breath for ≥ 10 minutes in the last week. Monthly income was classified into 4 groups: lowest, lower-middle, upper-middle, and highest quartiles. Education level was divided into 3 groups: participants who were uneducated or those who completed less than middle school were classified as the 'low' group, participants who graduated high school were classified as the 'middle' group, and participants who graduated junior college or college or graduate school were classified as the 'high' group. Region of residence was divided into 2 groups according to administrative district: urban (i.e., Seoul, Gyeonggi, Busan, Daegu, Incheon, Gwangju, Daejeon, Ulsan, and Sejong) and rural areas (i.e., Gangwon, Chungbuk, Chungnam, Jeonbuk, Jeonnam, Gyeongbuk, Gyeongnam, and Jeju). Participants were divided into 3 groups according to smoking status: nonsmokers, past smokers, and current smokers. The participants were asked to report their frequency of alcohol consumption and were divided into 4 groups accordingly: none, ≤ 1 time a month, 2-4 times a month, and ≥ 2 times a week. Obesity was measured using body mass index (BMI, kg/m^2). Participants with heights < 110 cm or weights < 30 kg were excluded from this study. BMI was categorized as < 18.5 (underweight), ≥ 18.5 to < 23 (normal), ≥ 23 to < 25 (overweight), ≥ 25 to < 30 (obese I), and ≥ 30 (obese II) based on the Asia-Pacific criteria following the Western Pacific Regional Office (WPRO) 2000. Subjective health status was divided into the following 3 groups: good, normal, and poor. Stress levels were divided into the following 4 groups: no stress, some stress, moderate stress, and severe stress. The amount of sleep was divided into the following categories: ≤ 5 hours per day, 6 hours per day, 7 hours per day, 8 hours per day, and ≥ 9 hours per day.
